# Blood Chemo-Profiling in Workers Exposed to Occupational Pyrethroid Pesticides

**DOI:** 10.3390/ijerph22050769

**Published:** 2025-05-13

**Authors:** Ohoud O. Sufyani, Magbool E. Oraiby, Ibraheem M. Attafi, Elsiddig Noureldin, Ommer Dafallah, Yahya A. Hobani, Sultan Qumayi, Ahmad Sahly, Yasser Majrabi, Ali Maashi, Abdulaziz A. Almane, Zaki M. Eisa, Abdullah Alaamri, Waheed Mohammed, Ahmed M. Hakami, Mohammed A. Attafi, Ibrahim A. Khardali, Ala’udin Hakami, Ebraheem Alkhyat, Mari H. Alnashri

**Affiliations:** 1 Central Blood Bank, Jazan Health Cluster, Jazan 45142, Saudi Arabia; oosufyani@moh.gov.sa; 2 Forensic Toxicology Services, Ministry of Health Branch in Jazan region, Jazan 45142, Saudi Arabia; moraiby@moh.gov.sa (M.E.O.); ahmahakami@moh.gov.sa (A.M.H.); mattafi@moh.gov.sa (M.A.A.); ikhardali@moh.gov.sa (I.A.K.); 3 South Al-Qunfudah General Hospital, Makkah Health Cluster, Makkah 21912, Saudi Arabia; mhalnashri@moh.gov.sa; 4 Weqaa Center Laboratories, Division of Disease Vectors, Jazan 45142, Saudi Arabiae18373@weqaa.gov.sa (A.A.A.); 5 Executive Management of Community and Public Health, Jazan Health Cluster, Jazan 45142, Saudi Arabia; yhobani@moh.gov.sa; 6 Jazan Health Cluster, Jazan 45142, Saudi Arabia; squmayi@moh.gov.sa (S.Q.); ymajrabi@moh.gov.sa (Y.M.); abmaashi@moh.gov.sa (A.M.); 7 Public Health Authority, Jazan 45142, Saudi Arabia; zmomar@pha.gov.sa (Z.M.E.); aialamri@pha.gov.sa (A.A.);; 8 Jazan Municipality, Jazan 45142, Saudi Arabia; aalhakami@momah.gov.sa (A.H.); alkhayat@momah.gov.sa (E.A.)

**Keywords:** workplace exposure, insecticide, metabolic biomarkers, GCMS analysis

## Abstract

This study investigates the effect of occupational exposure to pyrethroid insecticides on the blood chemo-profiles of workers in the Jazan region. This study was conducted to examine this issue, and workers were divided into exposure groups based on how long they had been employed—from one to two years to more than eight years. Blood samples were analyzed to determine their hematological and biochemical parameters, and their chemo-profiles were assessed by GCMS analysis. Workers exposed for 8+ years had a 3.7 times higher risk of chronic diseases than those exposed for 1–2 years (*p* < 0.01). Prolonged exposure to pyrethroid pesticides at work is linked to significant changes in blood chemical profiles. While gamma-glutamyl transferase (GGT) levels (*p* < 0.05) were rather increased by extended exposure times, albumin levels (*p* < 0.05) showed a significant decrease. These findings suggest re-evaluating and improving workplace safety practices to protect workers from extended pyrethroid exposure.

## 1. Introduction

As agriculture expands globally, farmers increasingly use insecticides to protect their crops and boost yields. This trend has unfortunately created workplace hazards: insecticide poisoning among agricultural workers [1]. Pesticides were the most often involved substance in human exposures in 2022 according to the 2022 NPDS Annual Report, which provided data on exposure cases reported to US poison centers [2]. Pesticide exposures accounted for 3.69% of the total number of single-substance exposures (3.53% in the pediatric population (≤5 years) and 4.61% in adults (≥20 years)).

Among the often used common pesticides are pyrethroids like deltamethrin, cyphenothrin, D-tetramethrin, lambdacyhalothrin, D-phenothrin, permethrin, niclosamide, cyfluthrin, and allethrin and organophosphates like fenitrothion. Although these compounds are rather effective at regulating pests, their acute toxicity in animals is the most evident [3,4]. They can also cause liver enlargement and a rise in liver enzyme activity and increase susceptibility to a range of diseases, including respiratory problems, skin disorders, and neurological effects of individuals who are exposed to high levels of these chemicals for long durations [5,6,7,8]. Since they are harmful and might have different effects on different health aspects, these substances endanger occupational workers.

Out of all the pyrethroids, only permethrin has been identified as potential or weak carcinogen by the US EPA; its mutagenicity has been assessed to be very low [5]. According to a report from the WHO [9], no laboratory research on human beings has revealed any carcinogens. Most of the 573 cases of acute pyrethroid primarily poisoning recorded in China between 1983 and 1988 came from occupational or unintentional exposure. Following the insecticide’s injection into air-conditioning ducts, a severe form of unintentional pyrethroid poisoning was recorded. The affected individuals complained of dyspnea, nausea, headache, and irritability [10,11]. While sensorimotor polyneuropathy in the lower extremities and vegetative nervous disorders, like paroxysmal tachycardia, increase heat sensitivity and reduce exercise tolerance related to circulatory dysfunction [11,12], the chronic sequelae of pyrethroid exposure include cerebro-organic disorders.

Repeated exposure has been linked to alterations in blood parameters, increased oxidative stress, and disrupted metabolic processes [11,13,14,15]. Analyzing these biomarkers using gas chromatography–mass spectrometry (GC-MS) analysis is therefore crucial, as it provides the sensitive and reliable detection of subtle changes in the biochemical profile that reflect the body’s response to pesticide exposure [16,17]. This precise monitoring enables the development of effective policies, implementing appropriate safety practices, establishing protective laws and regulations, and designing targeted therapeutic interventions, all important steps toward minimizing the harmful effects of these compounds on human health.

There have been various studies conducted to evaluate how pesticides affect hematological parameters. For example, the pyrethroids deltamethrin and permethrin have been linked to oxidative stress and alterations in red blood cell (RBC) count, hemoglobin levels, and white blood cell (WBC) count [18,19,20,21]. Fenitrothion, an organophosphate, has been linked to anemia and leukocytosis in those who have been exposed to the insecticide at work [22]. The aforementioned differences in blood markers indicate that long-term pesticide exposure causes immunological issues and systemic harm. Moreover, workers exposed to organophosphates and pyrethroids showed lower antioxidant enzyme activity [23,24]. These findings show that oxidative stress may be involved in the pathophysiology of pesticide poisoning, resulting in cell damage and inflammation.

Furthermore, exposure to multiple pesticides can lead to synergistic toxicity, where the combined effect is greater than the sum of individual effects, as well as cumulative effects, which refer to the gradual buildup of biological damage or physiological changes over time, even if the chemicals are cleared from the body rather than stored [25]. For example, the combination of deltamethrin and chlorpyrifos exhibited synergistic inhibition effects of catalase activity in the rat brain [26], and co-exposure to deltamethrin and imidacloprid produced synergistic neurotoxicity in adult zebrafish [27]. These findings highlight the importance of studying cumulative pesticide effects to better understand human health risks. Accordingly, this study aims to characterize blood chemo-profiles and identify specific metabolites as biomarkers of pyrethroid exposure, thereby providing a basis for predicting adverse outcomes and guiding preventive actions and risk assessments.

## 2. Materials and Methods

This research was conducted with the participation of a total of 250 male volunteers who were between the ages of 20 and 60 and who worked with pesticides in the Jazan region of Saudi Arabia. The use of a questionnaire allowed for the collection of a variety of information. The main sections of the questionnaire and consent form were study aims, risks and outcomes, and the participants’ demographic information (age, sex, education, and residence); occupational history (job, total years of pesticide work, particular job tasks, types of pesticides handled, frequency of pesticide application (days per week), average hours of exposure per day, and seasonal variations in work intensity); spraying methods (larvae, vacuum, etc.); health status (current and chronic diseases and medication use); and other data including household pesticide use, diet, and smoking. Workers handled pyrethroid substances including deltamethrin, cypermethrin, fenitrothion, cyphenothrin, D-tetramethrin, lambdacyhalothrin, D-phenothrin, cyfluthrin, allethrin, and permethrin. Usually during periods of maximum use, workers exposed to these substances applied the treatments 2–3 times weekly. On average, daily exposure lasts 4 to 6 h on application days. Larvae and vacuum spraying are the two spraying techniques used. Participants in this study had exposure intervals that ranged from one to two years, three to five years, six to eight years, and even more than eight years (exposure intervals: 1–2, 3–5, 6–8, more than 8 years).

Blood samples were collected for two analyses. Hematological and biochemical parameters were evaluated across four exposure duration groups (1–2, 3–5, 6–8, and 8+ years) at the Central Blood Bank, Jazan Health Cluster, Jazan, Saudi Arabia, while blood chemo-profiles were analyzed using liquid-liquid extraction followed by gas chromatography–mass spectrometry (GC-MS). Samples were prepared using the procedure described by Latif et al. (2012) [28], with a few modifications. An amount of 1 mL of whole blood was placed into a screw-capped vial that had a capacity of 20 mL. An internal standard of one microgram per milliliter of triphenylphosphine (TPP) was added to the blood. Immediately following that, 1 mL of methanol was added to the mixture, and it was vortexed for a duration of 2 min. After adding 10 mL of extraction solvent (n-hexane:acetone, 9:1 *v*/*v*) to the mixture, 2 grams of anhydrous sodium sulfate were introduced. The bottles were then subjected to sonication for 2 min to facilitate extraction. Following this, the bottles were centrifuged for a period of 5 min at a speed of four thousand revolutions per minute. The organic layer was then filtered into clean 12 mL tubes using a 0.45 µm syringe filter. The filtrate was evaporated to dryness under nitrogen gas. The residues were then reconstituted in 200 µL of methanol and transferred to 2 mL GCMS vials with 300 µL of insert.

A gas chromatograph–mass spectrometer (GCMSQP-2010 ultra, Shimadzu, Japan) was utilized in order to carry out the GC. It was developed in accordance with the description that was supplied by Hassan et al. (2014) [29] with a few modifications. For the purpose of chromatographic separation of components, a capillary column manufactured by Thermo Scientific (Thermo Scientific, Melbourne, Australia) and designated as TR-5 MS was utilized. The interior diameter of the column measured 35 mm, and its thickness measured 25 μm. The column measured thirty meters in length. One milliliter per minute was found to be the optimal flow rate for the helium carrier gas, according to the findings of the study. In order to inject the sample extract, which had a volume of 2 µL, into the injection port, a splitless mode that was heated to 260 °C was utilized. For 1 min, the temperature in the oven was kept at 70 °C, and then it gradually increased to 210 °C at a rate of 30 °C per minute, with a hold interval of 2 min in between each increment. In the end, the ramping rate was increased to 290 °C at a rate of 20 degrees per minute, and there was a holding period of 15 min. A temperature of 70 °C was chosen for the oven’s beginning temperature for a period of 1 min. It was necessary to make use of the electron ionization (EI) source in addition to the scan mode that was contained within the mass spectrometer in order to run it. The electron energy was 70 eV and the temperature of the interface was maintained at 290 °C, while the temperature of the ion source was maintained at 230 °C.

Data analysis was performed using R Statistical Software (version 1.0.2) to verify the accuracy of the library entries. After this validation step, we proceeded with data analysis and presentation using Python (version 3.11.6) and Excel (version 2503). Data were grouped by exposure groups and compounds, followed by the calculation of descriptive statistics (mean, SEM, and occurrences). The Shapiro–Wilk test revealed non-normal distribution (*p* < 0.001), which, combined with unequal sample sizes, necessitated non-parametric analysis via the Kruskal–Wallis rank sum tests (significance: *p* < 0.05). Relationships between variables were further examined using linear regression models and Pearson’s correlation coefficients. For significant compounds, mean peak areas were compared and 95% confidence intervals were calculated.

## 3. Results

This study investigated the relationship between the frequency of chronic diseases and the duration of pesticide exposure in 250 workers. The age distribution of the participants was as follows: 20–30 (62), 31–40 (101), 41–50 (62), and 51–60 (25). The participants ranged in age from 20 to 60. Most frequently the participants were between the ages of 31 and 40, but participants also fell in the 20–30 and 41–50 age ranges. The four groups of exposure times were 1–2 years (n = 52), 3–5 years (n = 44), 6–8 years (n = 26), and >8 years (n = 128). Although most participants had either secondary or tertiary education, a small number had only finished primary school.

**Figure 1:**
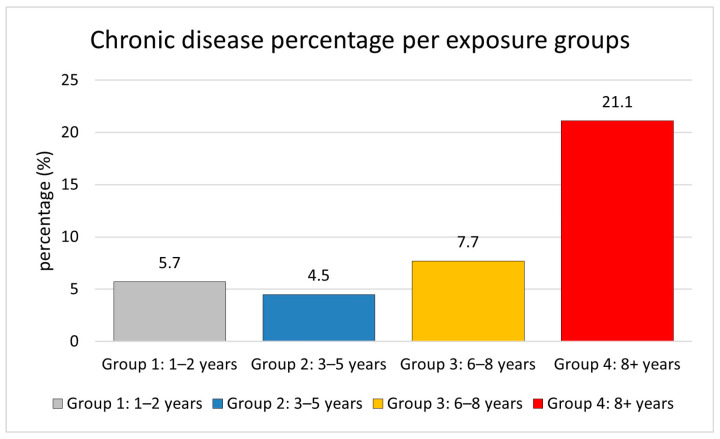
Association between occupational exposure duration and chronic disease risk. Bar plot showing the prevalence of chronic diseases across different exposure duration groups. The *x*-axis represents exposure groups (Group 1: 1–2 years, Group 2: 3–5 years, Group 3: 6–8 years, and Group 4: 8+ years), while the *y*-axis shows the percentage of disease prevalence (%). A significant increase in chronic disease prevalence is observed in Group 3 (7.7%) and Group 4 (21.1%) compared to Group 1 (5.7%), representing a 3.7-fold increase in risk (*p* = 0.00553, Kruskal–Wallis test).

Longer exposure increases the risk of chronic diseases, including diabetes, hypertension, respiratory disorders, and cardiovascular disease. The likelihood of developing chronic disorders increases with longer exposure durations, with rates of 5.7% for one to two years, 4.5% for three to five years, 7.7% for six to eight years, and 21.1% for more than eight years, as shown in Figure 1. This indicates that workers in exposure Group 4 (8+ years) have a roughly 3.7-fold higher risk of chronic diseases compared to those in exposure Group 1 (1–2 years). Chronic diseases were relatively infrequent in the shorter exposure groups but increased significantly in workers with longer exposure periods.

The trend analysis revealed a significant association between exposure duration and chronic disease prevalence. This demonstrates the substantial increase in chronic disease prevalence associated with longer exposure durations, particularly in Group 4, where the prevalence is approximately 3.7 times higher than in Group 1. Starting with 5.7% in the 1–2 years group and 4.5% in the 3–5 years group, rising to 7.7% in the 3–5 years group, and then reaching 21.1% in the 8+ years group, the frequency of chronic diseases exhibited a steady increase with longer exposure periods. This trend shows a significant link between chronic disease risk and exposure length; workers with more than 8 years of exposure show particularly greater susceptibility to disease.

### 3.1. Blood Parameter Analysis Results

Hematological and biochemical parameters were assessed across four exposure duration groups (1–2, 3–5, 6–8, and 8+ years). Complete blood counts and comprehensive biochemical profiles were obtained for all participants, with results summarized in Table 1.

**Figure 2:**
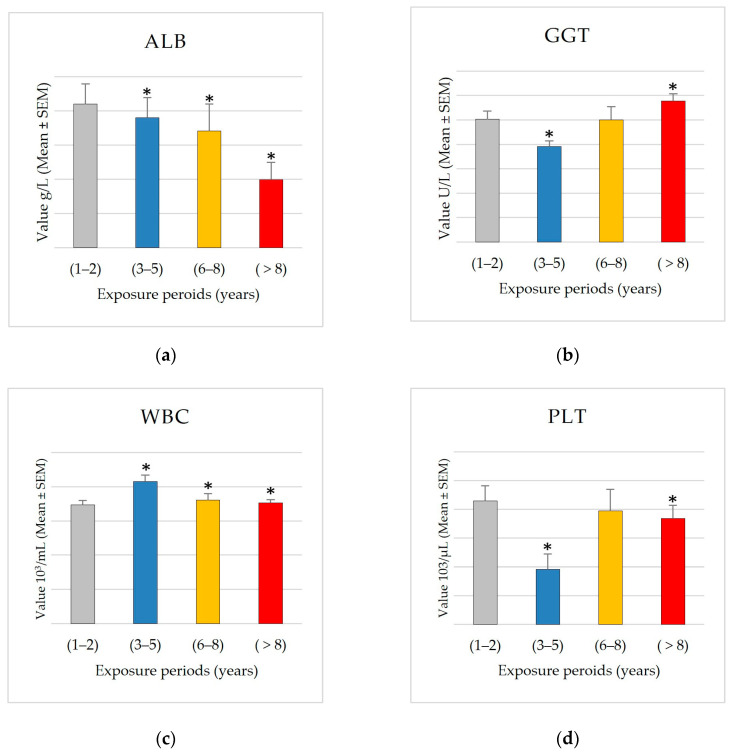
Blood parameters across different exposure periods. The bar plot reveals significant differences in blood parameters across exposures. Each panel represents one parameter listed as follows: (**a**) albumin (ALB); (**b**) gamma-glutamyl transferase (GGT); (**c**) white blood cell count (WBC); (**d**) platelet count (PLT). The *x*-axis represents exposure groups (Group 1: 1–2 years, Group 2: 3–5 years, Group 3: 6–8 years, and Group 4: 8+ years), while the *y*-axis shows the mean value of each blood parameter group. Statistical analysis: Kruskal–Wallis test indicates significant overall differences between groups (*p* < 0.05), followed by Dunn’s post hoc test for pairwise comparisons against Group 1 (1–2 years). Asterisks (*) indicate significant differences compared to Group 1 (*p* < 0.05).

The analysis of blood parameters across different exposure durations revealed significant alterations in several parameters. The bar plot for significant parameters (Figure 2) demonstrates the changes in parameter levels with the increase in exposure duration.

Albumin (ALB) levels exhibited a significant declining trend with increased exposure duration (*p* = 0.003). The mean albumin (ALB) levels showed a significant decrease in the 8+ years group (37.95 ± 0.50 g/L) compared to the 1–2 years group (40.20 ± 0.59 g/L), representing a consistent downward trend across exposure categories. Based on the correlation analysis between exposure period and blood parameters, the Pearson correlation analysis revealed a significant negative correlation between exposure duration and serum albumin (ALB) levels (r = −0.952, *p* = 0.048), indicating a strong inverse relationship between exposure time and albumin concentration. This suggests a potential decline in liver synthetic function with prolonged exposure. With a *p* = 0.0002, gamma-glutamyl transferase (GGT) showed the most notable change among each of the parameters. From 50.35 ± 3.26 U/L in the 1–2 years group to 57.83 ± 2.91 U/L in the 8+ years group, GGT levels showed an increasing tendency with respect to exposure time; there was a clear dip in the 3–5 year group (39.05 ± 2.38 U/L). Additionally, statistically significant changes in white blood cell count (WBC) (*p* = 0.016) and platelet count (PLT) (*p* = 0.032) were found.

RBC, HGB, HCT, MCV, MCH, MCHC, TP, ALPI, AST, and ALTI were among the other blood parameters that did not approach statistically significant differences across exposure groups (*p* > 0.05). This indicates that there are more subtle alterations in these markers between groups. Nevertheless, for all of the exposure durations, these metrics remained within the guidelines of their respective reference ranges.

### 3.2. Blood Chemo-Profiling Analysis

The GCMS study identified compounds displaying significant differences across exposure groups. Overall, the findings show that 1080 different chemicals were found among all the groups. Investigating group distributions, unique compounds, and compound area trends allowed us to identify the most important trends and variations among the exposure groups. Indicating a greater diversity of chemicals with longer exposure times, Group 4 (8+ years) had the most samples (127) and unique compounds (268). With 25 samples and 48 unique compounds, Group 3 (6–8 years) had the lowest compound identified range. Groups 1 (1–2 years) and 2 (3–5 years) had correspondingly modest sample numbers (52 and 44, respectively) and unique compounds (142 and 118, respectively). Group 4 (8+ years) contained the highest number of compounds, with significant unique compounds per group according to the intersection analysis; meanwhile, the intersections expose notable overlap between groups, especially between adjacent exposure length groups. This implies the formation of new compounds with extended exposure times as well as the persistence of some compounds over time.

**Figure 3:**
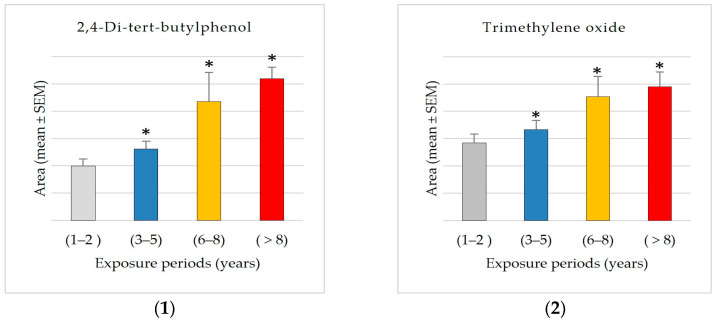

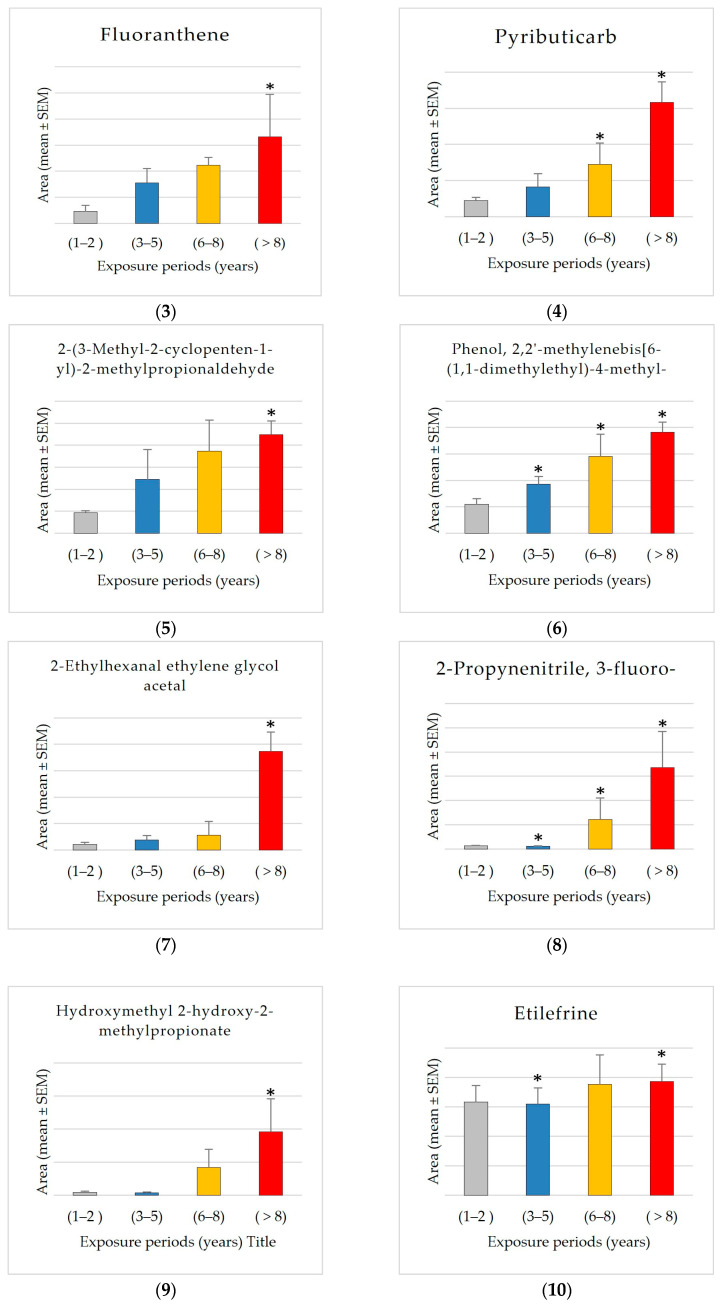

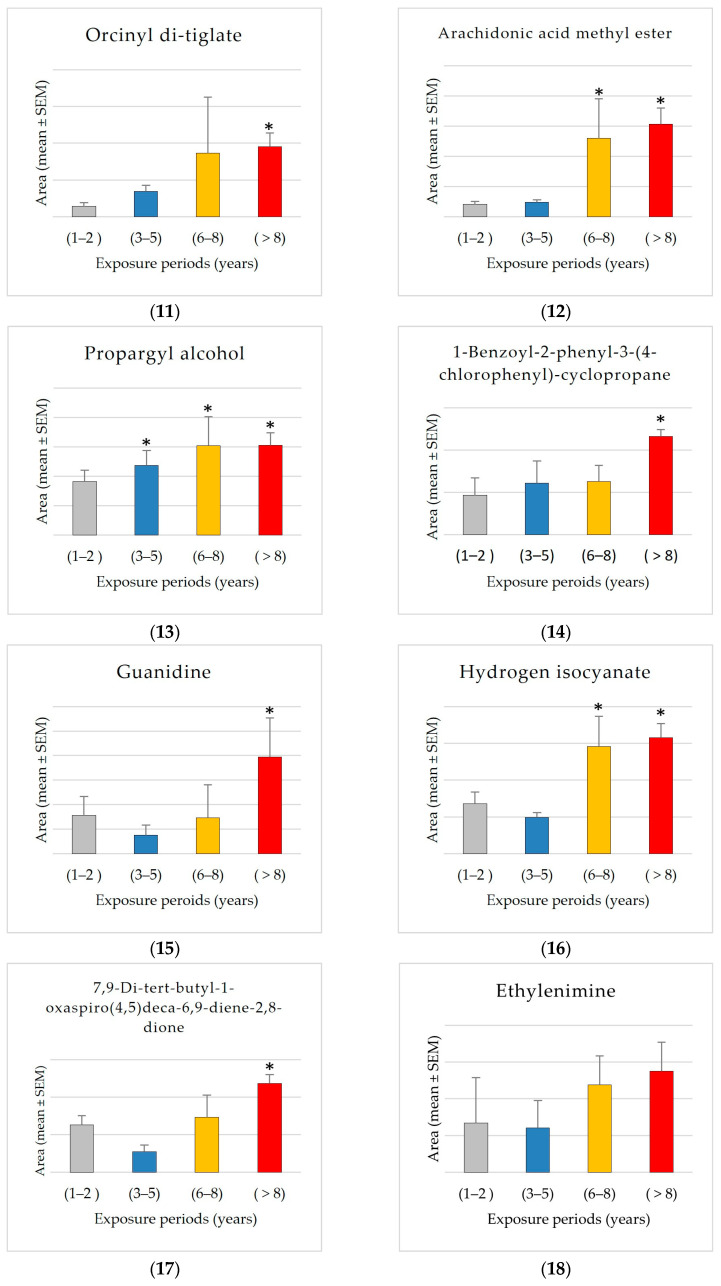

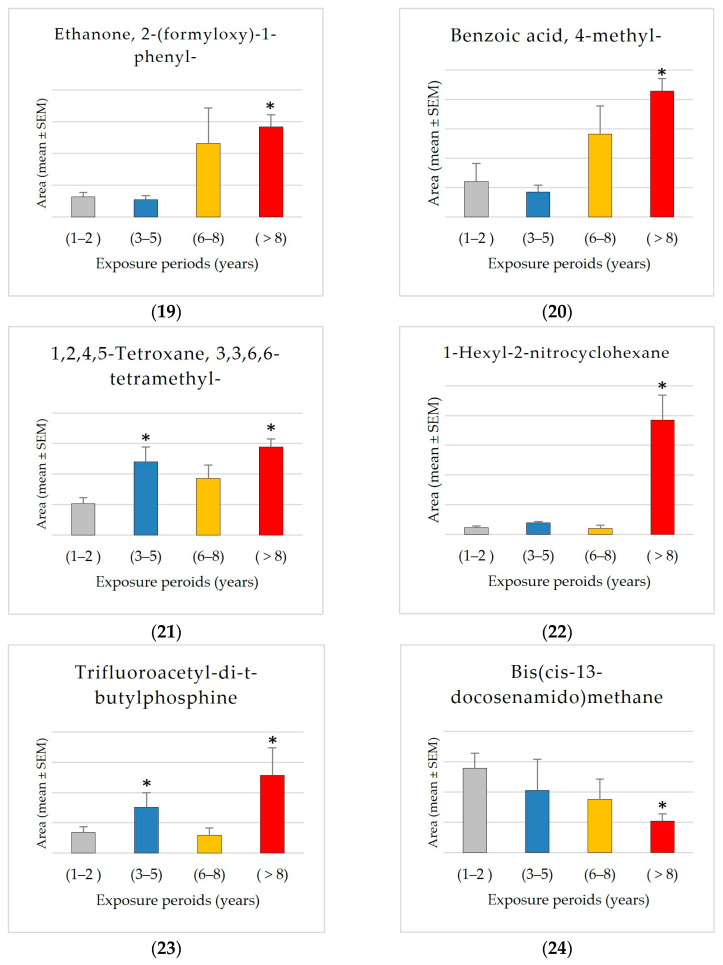
Exposure-related changes in common compound levels across different exposure periods. The bar plot reveals significant differences in common compounds across exposures. Each panel represents one compound listed as follows: (**1**) 2,4-Di-tert-butylphenol; (**2**) Trimethylene oxide; (**3**) Fluoranthene; (**4**) Pyributicarb; (**5**) 2-(3-Methyl-2-cyclopenten-1-yl)-2-methylpropionaldehyde; (**6**) Phenol, 2,2′-methylenebis [6-(1,1-dimethylethyl)-4-methyl-; (**7**) 2-Ethylhexanal ethylene glycol acetal; (**8**) 2-Propynenitrile, 3-fluoro-; (**9**) Hydroxymethyl 2-hydroxy-2-methylpropionate; (**10**) Etilefrine; (**11**) Orcinyl di-tiglate; (**12**) Arachidonic acid methyl ester; (**13**) Propargyl alcohol; (**14**) 1-Benzoyl-2-phenyl-3-(4-chlorophenyl)-cyclopropane; (**15**) Guanidine; (**16**) Hydrogen isocyanate; (**17**) 7,9-Di-tert-butyl-1-oxaspiro(4,5)deca-6,9-diene-2,8-dione; (**18**) Ethylenimine; (**19**) Ethanone, 2-(formyloxy)-1-phenyl-; (**20**) Benzoic acid, 4-methyl-; (**21**) 1,2,4,5-Tetroxane, 3,3,6,6-tetramethyl-; (**22**) 1-Hexyl-2-nitrocyclohexane; (**23**) Trifluoroacetyl-di-t-butylphosphine; (**24**) Bis(cis-13-docosenamido)methane. The *x*-axis represents exposure groups (Group 1: 1–2 years, Group 2: 3–5 years, Group 3: 6–8 years, and Group 4: 8+ years), while the *y*-axis shows the mean value of compound peak area. Statistical analysis: Kruskal–Wallis test indicates significant overall differences between groups (*p* < 0.05), followed by Dunn’s post hoc test for pairwise comparisons against Group 1 (1–2 years). Asterisks (*) indicate significant differences compared to Group 1 (*p* < 0.05).

In addition, 192 compounds were always present in all four exposure groups, which are the key compounds that persist during all exposure times. Out of the 192 prevalent compounds, 25 exhibited relationships with exposure times. Moreover, this study revealed compounds with both growing and declining trends between groups; for most compounds across exposure groups, the distribution showed clear increases in mean area (Figure 3). Among them, 1 showed negative connections, and 24 chemicals showed positive ones. Correlation coefficients between the average area of each compound and the exposure period were obtained in order to find compounds having significant correlations to exposure periods.

The values of common compounds were determined for four exposure groups: Group 1 (1–2 years), Group 2 (3–5 years), Group 3 (6–8 years), and Group 4 (8+ years). This study found significant exposure-related patterns in various common chemical components across the four exposure groups (1–2 years, 3–5 years, 6–8 years, and 8+ years). The compounds had both positive and negative associations with exposure periods, indicating systematic changes in chemical compositions over time (Figure 3).

The results showed significant exposure-related increases, with progressive increases observed from Group 1 (1–2 years) to Group 4 (8+ years) in the levels of 2-ethylhexanal ethylene glycol acetal (1600% increase), 1-hexyl-2-nitrocyclohexane (1562% increase), arachidonic acid methyl ester, pyributicarb (614% increase), 3-phenyl-6-(4-nitrophenyl)-4H-(1,2,3)triazolo(1,5-d)(1,3,4)oxadiazin-4-one (505% increase), 2-(3-methyl-2-cyclopenten-1-yl)-2-methylpropionaldehyde (380 increase), ethanone, 2-(formyloxy)-1-phenyl-(+350% increase), phenol, 2,2′-methylenebis [6-(1,1-dimethylethyl)-4-methyl- (250% increase), 1,2,4,5-tetroxane, 3,3,6,6-tetramethyl- (180% increase), 2,4-di-tert-butylphenol (159% increase), 1-benzoyl-2-phenyl-3-(4-chlorophenyl)-cyclopropane (150% increase), benzoic acid, 4-methyl- (148% increase), hydrogen isocyanate (132% increase), 7,9-di-tert-butyl-1-oxaspiro(4,5)deca-6,9-diene-2,8-dione (+87.66% increase), and trimethylene oxide (72% increase).

The findings also showed an exposure-related escalation, characterized by a consistent upward pattern in mean levels correlating with exposure duration, particularly between Group 1 (1–2 years) and Group 4 (8+ years) for 2-propynenitrile, 3-fluoro- (2344% increase), hydroxymethyl 2-hydroxy-2-methylpropionate (2234% increase), fluoranthene (650% increase), orcinyl di-tiglate (556% increase), trifluoroacetyl-di-t-butylphosphine (272% increase), guanidine (153% increase), ethylenimine (106% increase), propargyl alcohol (68% increase), and etilefrine (22% increase). The consistent rise in many compounds suggests that accumulation associated with exposure is prevalent. The results reveal a propensity for elevated levels with exposure; however, the variability within groups complicates the identification of statistically significant differences. Additional research with larger sample sizes may be necessary to confirm these trends.

In contrast, the compound bis(cis-13-docosenamido)methane is unique among the investigated compounds in that it shows a significant decrease in average detected area with increasing exposure times. Specifically, the average area measured in the shortest exposure group (1–2 years) is approximately 2.7 times greater than that observed in the group exposed for 8+ years. This negative trend may indicate that this compound accumulates more in shorter exposure groups. The significant reduction of approximately 62% from Group 1 to Group 4 suggests a possible exposure-related metabolic or environmental mechanism influencing the levels of bis(cis-13-docosenamido)methane.

### 3.3. The Relationship Between Common Compounds and Albumin Concentrations

**Figure 4:**
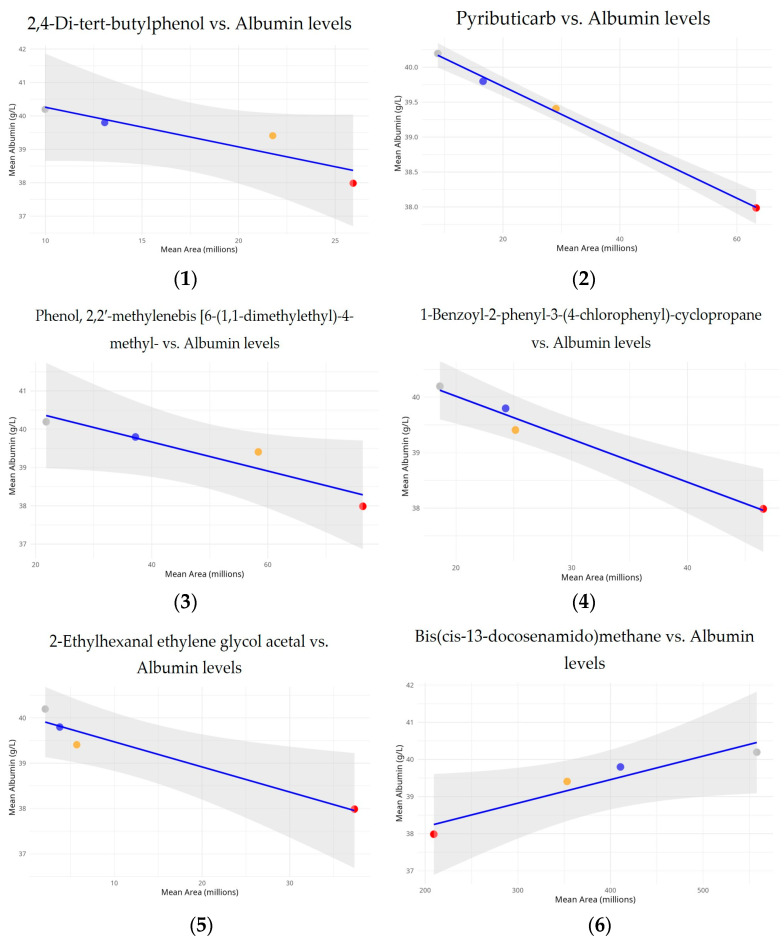
Relationship between common compounds and serum albumin levels across different exposure durations. Each panel represents one compound listed as follows: (**1**) 2,4-Di-tert-butylphenol; (**2**) Pyributicarb; (**3**) Phenol, 2,2′-methylenebis [6-(1,1-dimethylethyl)-4-methyl-; (**4**) 1-Benzoyl-2-phenyl-3-(4-chlorophenyl)-cyclopropane; (**5**) 2-Ethylhexanal ethylene glycol acetal; (**6**) Bis(cis-13-docosenamido)methane. Mean serum albumin concentrations (g/L) are plotted against mean compound areas (in millions) for four exposure groups, each represented by a different colored circle: gray for 1–2 years (n = 52), blue for 3–5 years (n = 44), orange for 6–8 years (n = 25), and red for 8+ years (n = 127). The blue line represents the linear regression fit, with the shaded area indicating the 95% confidence interval.

In order to determine if common compounds had significant effects on albumin concentrations, linear regression models were used to examine the relationship between common compound relative levels and albumin concentrations across the exposure groups (Figure 4).

The analysis reveals a strong and statistically significant negative relationship between albumin levels and pyributicarb (*p* = 0.001, R^2^ = 0.998), 1-benzoyl-2-phenyl-3-(4-chlorophenyl)-cyclopropane (*p* = 0.011, R^2^ = 0.977), and 2-ethylhexanal ethylene glycol acetal (*p* = 0.032, R^2^ = 0.937). The inverse relationship appears to be statistically significant, with a dose–response pattern across the exposure groups. The strong R^2^ value indicates that increased exposure to this compound significantly contributes to lower albumin levels.

This study also revealed a notable inverse connection between albumin levels and area measurement for phenol, 2,2′-methylenebis [6-(1,1-dimethylethyl)-4-methyl- (p = 0.057, R^2^ = 0.889), and 2,4-di-tert-butylphenol (*p* = 0.086, R^2^ = 0.836) across the analyzed exposure groups. Unlike other compounds, bis(cis-13-docosenamido)methane shows a positive connection with albumin levels (*p* = 0.050, R^2^ = 0.902). Calculated to be 0.950, the correlation value indicated a strong positive linear relationship. Although the slope’s *p*-value is slightly higher than the usual 0.05 threshold, it still suggests a tendency toward significance, especially given that this study is based on four distinct exposure groups.

## 4. Discussion

In 250 participants occupationally exposed to pesticides in the Jazan region of Saudi Arabia, this study examined the effects of pesticide exposure on blood parameters as well as the significant changes in blood chemo-profiles related to exposure periods.

The prevalence of chronic diseases and exposure time showed a notable correlation according to the trend study. Workers with 8 or more years of exposure had a 3.7-fold higher risk of chronic diseases than those with 1–2 years of experience, therefore proving that the longer the exposure, the higher the risk of chronic health problems exists. This tendency is in agreement with previous studies showing that total occupational hazard exposure—including pesticides—highly increases the incidence of chronic health problems. Research on pesticide exposure workers has revealed that longer exposure times increase the risk of respiratory and neurological illnesses [30]. In this study, the observed risk increase after six years suggests a threshold effect, accelerating disease development by overwhelming the body’s compensatory systems. This study population has chronic diseases at a level of 10.80%, which is consistent with occupational cohort data. However, demographics, intensity, and exposure type may vary. Studies show that long-term pesticide exposure increases the risk of chronic diseases such diabetes [31,32]. These findings emphasize the importance of improving workplace preventative actions, particularly for long-term exposed workers. Early intervention, exposure reduction, and health monitoring lower chronic illness risk.

Additionally, the results indicate a correlation between alterations in some blood parameters and prolonged exposure durations. The most alarming results originated from GGT and albumin levels, indicating potential hepatic damage due to prolonged exposure. GGT exhibits an increasing trend relative to exposure duration, particularly in the 8+ age demographic, whereas ALB demonstrates an overall decreasing trend with extended exposure time.

Our finding that albumin levels were significantly lower after 8+ years of exposure compared to 1–2 years aligns with established literature on pesticide exposure effects. Palaniswamy et al. (2021) [33] reported reduced serum albumin in agricultural workers chronically exposed to organophosphates, while Tarhoni et al. (2008) [34] showed that prolonged exposure to pesticides inhibits liver enzymes involved in protein synthesis. These biochemical alterations may explain the observed reduction in albumin production, as the liver is the primary site of albumin synthesis. These effects appear to involve either direct hepatocellular damage impairing protein synthesis or increased metabolic demands enhancing protein catabolism. Both studies revealed a dose–response relationship where longer exposure correlates with decreasing albumin levels. This consistent pattern across diverse populations suggests that albumin may serve as a sensitive biomarker for monitoring chronic pesticide exposure, potentially detecting subclinical hepatic effects before conventional markers indicate severe liver dysfunction.

Moreover, our findings regarding increased GGT levels with prolonged pesticide exposure support previous work by Hernández et al. (2013) [35]. They linked pesticide exposure to biochemical signs of liver damage, including elevated GGT levels. This increase in GGT, which is a sensitive marker of hepatobiliary injury, may reflect ongoing hepatic stress and injury resulting from chronic exposure to pesticides. Similarly, Kumar et al. (2014) [36] demonstrated that increased persistent organic pollutants, including pesticides, correlate with elevated GGT and altered albumin levels. Furthermore, Sun et al. (2019) [37] showed that impaired albumin function, including its binding, transport, and antioxidant properties, can serve as an early marker of liver dysfunction even when albumin levels remain within normal ranges.

These findings indicate that pesticide exposure may impair hepatic detoxification, leading to oxidative stress and altered liver function, reflected by changes in both GGT and albumin. The observed elevation of GGT indicates hepatobiliary injury, while alterations in albumin levels point toward compromised protein synthesis. This aligns with our findings regarding albumin alterations and the GGT elevation we observed. Based on our findings, we propose that both markers appear particularly valuable as sensitive indicators of prolonged pesticide exposure effects. This observation warrants further investigation that should first account for potential confounding variables, second evaluate its utility as a surveillance tool in occupational health monitoring programs, and finally explore possible therapeutic interventions for affected individuals.

The GCMS results analysis demonstrated that Group 4 (8+ years) had the most distinct compounds, showing that prolonged exposure complicates the chemical profile. It may imply cumulative effects or secondary compounds generated over time. The data also show dynamic compound abundance variations throughout exposure periods, with significant correlations and fold changes suggesting biomarkers or exposure-related effects, including 2,4-di-tert-butylphenol, trimethylene oxide, pyributicarb, phenol, 2,2′-methylenebis [6-(1,1-dimethylethyl)-4-methyl-, 1-benzoyl-2-phenyl-3-(4-chlorophenyl)-cyclopropane, 2-(3-methyl-2-cyclopenten-1-yl)-2-methylpropionaldehyde, 2-ethylhexanal ethylene glycol acetal, hydrogen isocyanate, benzoic acid, 4-methyl-, ethanone, 2-(formyloxy)-1-phenyl-, 7,9-di-tert-butyl-1-oxaspiro(4,5)deca-6,9-diene-2,8-dione, 3-phenyl-6-(4-nitrophenyl)-4H-(1,2,3)triazolo(1,5-d)(1,3,4)oxadiazin-4-one, 1-hexyl-2-nitrocyclohexane, arachidonic acid methyl ester, and 1,2,4,5-tetroxane, 3,3,6,6-tetramethyl-. The results showed that exposure increased the previously mentioned compounds, especially after 8 years. A significant exposure-related accumulation pattern suggests it may be a marker for metabolic alterations. It may also indicate physiological or environmental variables affecting its accumulation. More research is needed to confirm these findings and use it as an exposure-related biomarker and metabolic monitor. All these compounds can behave as reactive intermediates impacting redox equilibrium, metabolism, or enzyme efficiency.

Interestingly, propargyl alcohol levels increased significantly from 1–2 to 3–5 years of exposure. This chemical is widely utilized as a solvent and intermediary in pesticide formulations. Its high reactivity and capacity for oxidative stress generation have been observed and proven to cause adenomas in toxicological studies [38]. Furthermore, the data show that trimethylene oxide, a chemical that appears to induce atherosclerosis, increased by 72% from exposure Group 1 to Group 4. Trimethylene oxide has been linked to increased plaque in the aorta [39]. 2,4-Di-tert-butylphenol and pyributicarb have toxicity characteristics that distinguish them from other detected compounds with significant associations. 2,4-Di-tert-butylphenol disrupts the endocrine system [40], and pyributicarb alters metabolism [41]. This study also found significant temporal trends in hydrogen isocyanate levels across exposure periods, suggesting cumulative effects. Chemically reactive hydrogen isocyanate is used in industry and as a pesticide byproduct. The level rise with prolonged exposure may indicate bioaccumulation. Since isocyanates form persistent protein adducts, they persist in biological tissues longer [42,43]. Chronic isocyanate exposure can cause asthma and hypersensitivity pneumonitis [42,44]. These findings emphasize the significance of monitoring and limiting exposure to these compounds and the need for additional studies into long-term health effects, particularly in occupationally exposed populations. While bis(cis-13-docosenamido)methane exhibited a significant positive correlation, pyributicarb, 1-benzoyl-2-phenyl-3-(4-chlorophenyl)-cyclopropane, and 2-ethylhexanal ethylene glycol acetal showed significant negative correlations with albumin levels. The positive relationship with bis(cis-13-docosenamido)methane could suggest a compensating or protective biological effect on albumin levels at given exposure levels, thereby requiring more investigation.

This study’s small sample size in some demographic categories, as well as other potential confounding factors, limit its ability to fully represent the complexity of chemical exposure. Further research is needed to confirm these findings and assess the health effects of these compounds, particularly for vulnerable groups.

## 5. Conclusions

In conclusion, this study found significant links between pesticide exposure and health markers. Workers with 8+ years of exposure had a higher risk of chronic diseases than those with 1–2 years of exposure. Several compounds increased while some decreased with exposure length. This study reveals chemical exposure trends throughout time. The observed trends may be pesticide exposure biomarkers, but higher sample sizes are needed to confirm this. This study emphasizes the necessity for health monitoring and targeted exposure reduction.

## Figures and Tables

**Table 1:** Hematological and biochemical parameters across different exposure groups.

Parameter	Study Groups (Exposure Period)			
	1–2 Years	3–5 Years	6–8 Years	8+ Years
WBC (×10^3^/mL)	6.93 ± 0.26	8.30 ± 0.39	7.23 ± 0.36	7.05 ± 0.19
RBC (×10⁶/μL)	5.67 ± 0.10	5.73 ± 0.09	5.55 ± 0.07	5.45 ± 0.08
HGB (g/dL)	14.61 ± 0.29	14.85 ± 0.17	14.74 ± 0.19	14.19 ± 0.22
HCT (%)	45.67 ± 0.75	45.43 ± 0.59	45.49 ± 0.64	43.93 ± 0.66
PLT (×10^3^/μL)	345.92 ± 10.58	298.20 ± 10.61	338.88 ± 14.95	333.68 ± 9.19
MCV (FL)	81.59 ± 1.20	80.51 ± 1.10	82.99 ± 1.11	79.43 ± 1.14
MCH (PG)	25.68 ± 0.44	26.05 ± 0.44	26.68 ± 0.39	25.92 ± 0.49
ALB (g/L) *	40.20 ± 0.59	39.80 ± 0.59	39.41 ± 0.79	37.95 ± 0.50 *
GGT (U/L) *	50.35 ± 3.26	39.05 ± 2.38	49.42 ± 5.23	57.83 ± 2.91
TP (g/L)	76.84 ± 0.89	74.94 ± 1.11	74.47 ± 1.45	74.76 ± 0.87
ALPI (U/L)	86.40 ± 3.72	83.91 ± 3.41	83.76 ± 5.95	90.54 ± 2.27
AST (U/L)	25.56 ± 1.15	26.89 ± 1.88	23.36 ± 1.31	26.03 ± 0.79
ALTI (U/L)	47.21 ± 2.64	45.02 ± 5.66	46.64 ± 5.15	47.39 ± 1.76

Note: values are presented as mean ± standard error of the mean (SEM). WBC: white blood cell count; RBC: red blood cell count; HGB: hemoglobin; HCT: hematocrit; MCV: mean corpuscular volume; MCH: mean corpuscular hemoglobin; MCHC: mean corpuscular hemoglobin concentration; PLT: platelet count; ALB: albumin; GGT: gamma-glutamyl transferase; TP: total protein; ALPI: alkaline phosphatase; AST: aspartate aminotransferase; ALTI: alanine aminotransferase. Exposure groups are categorized as 1–2 years (n = 52), 3–5 years (n = 44), 6–8 years (n = 25), and 8+ years (n = 127). * Indicates parameters with statistically significant differences between groups (*p* < 0.05).

## Data Availability

All relevant details are included in the article.
